# Association between watching *wide show* as a reliable COVID-19 information source and preventive behaviors: A nationwide survey in Japan

**DOI:** 10.1371/journal.pone.0284371

**Published:** 2023-04-11

**Authors:** Keisuke Kuwahara, Mio Kato, Hirono Ishikawa, Tomohiro Shinozaki, Takahiro Tabuchi

**Affiliations:** 1 Teikyo University Graduate School of Public Health, Itabashi-ku, Tokyo, Japan; 2 Tokyo University of Science, Katsushika-ku, Tokyo, Japan; 3 Cancer Control Center, Osaka International Cancer Institute, Chuo-ku, Osaka, Osaka, Japan; St John’s University, UNITED STATES

## Abstract

**Introduction:**

Current pandemic prompted a surge in the television (TV) news watching. However, its influence is poorly understood. In Japan, *wide show*, a major genre of soft news TV programs, broadcasted COVID-19 for long hours, and was pointed out that it broadcasted COVID-19 sensationally, arousing fear and anxiety, and that it criticized individuals gathering in closed places. Thus, *wide show* may promote preventive behaviors but also produce fear or anxiety and aggressiveness towards others not engaging in preventive behaviors. We examined this issue using large-scale nationwide data.

**Methods:**

We analyzed the cross-sectional data of 25,482 individuals from the Japan COVID-19 and Society Internet Survey conducted in 2020. Participants reported the type of COVID-19 information sources including TV news and *wide show*, and their trustworthiness. We calculated multivariable-adjusted prevalence ratios (PRs) of engaging in recommended preventive behaviors strictly (defined as always engaging in hand washing, mask wearing, and attempting to keep physical distancing) and alerting others not engaging in preventive behaviors, respectively.

**Results:**

About 72.4% of the participants obtained information from TV news with reliance, while corresponding values were 50.3% for *wide show*. Overall, 32.8% engaged in recommended preventive behaviors strictly, and 9.6% alerted others. Watching *wide show* both with and without reliance were significantly associated with alerting others (adjusted PRs: 1.48 and 1.34, respectively) but not associated with preventive behaviors. Watching TV news was neither associated with strict preventive behaviors nor alerting others.

**Conclusion:**

Watching TV news and *wide show* was not associated with strict preventive behaviors; watching *wide show* was associated with only alerting others. Although causality is unclear, actions may be needed for TV stations broadcasting *wide show* to understand own influences on society in a timely manner amid the health emergencies.

## Introduction

For prevention and control of COVID-19, the public’s reactions are crucial [1]. The early data suggested that prevalence of recommended preventive behaviors, such as hand washing, mask wearing, and physical distancing, increased after the outbreak of COVID-19, then, gradually declined [2, 3]. Since behavioral changes are driven by threat perception and social norms that are influenced by various information sources [1], it is important to gain a better understanding of the impact of these sources on the public reactions for crisis management.

Previous studies amid the present pandemic have assessed the association of information sources, including government [4, 5], official information [6], television (TV) [7, 8], social media [4, 6, 9, 10], online news [9, 10], and newspapers [7] with behavioral outcome. However, some issues need further investigation. First, during this pandemic, an increase of TV news watching was observed in many countries [11], including in Japan [12], possibly to gain the latest information regarding COVID-19. Although this increase suggests a greater influence of TV on the public’s behavior, evidence on this issue amid the present pandemic is sparse [7, 8]. Second, in Japan, soft news TV programs called *wide show*, typically a one-and-a-half to two-hour TV program (similar to “The Daily Show” and “Good Morning America” in the US) broadcasted almost daily in the morning, at noon, and in the evening, are popular and broadcasted COVID-19 information for long hours [13]. As the sensationalism in TV news has been identified as public health issues [14], the *wide show* programs have been indicated as broadcasting COVID-19 sensationally [15] and arousing fear or anxiety [15, 16]. Another unique aspect of the *wide show* programs is that they entail comments and discussions by newscasters and commentators who are not health or medical professionals, such as entertainer, famous people, etc. [17] and that they criticize others, for example, individuals gathering in closed places amid this pandemic [18]. Thus, while these *wide show* programs may promote preventive behaviors, they may also produce excessive fear or anxiety and aggressiveness toward others who do not adhere to recommended preventive behaviors [19]. This concern is supported by their long-time exposure from the cultivation theory [14]. However, scientific research was not conducted for *wide show* programs amid this pandemic. Third, the existing analyses on TV have not adjusted for other information sources [7, 8], which may affect behaviors. Lastly, few studies on this topic have used large-scale nationwide data [4, 19], which can help us understand the public’s response at the national level.

Therefore, we assessed the association of watching TV news and *wide show* as COVID-19 information sources with the recommended preventive behaviors and alerting using the large-scale nationwide data.

## Methods

### Study design and settings

We conducted the nationwide online cross-sectional study among residents in Japan. We used the baseline data from the Japan COVID-19 and Society Internet Survey (JACSIS). As described previously [20], in 2020, 224,389 panelists aged 15 to 79 years were invited to participate in the survey; they were selected based on random sampling stratified by age, sex, and prefecture covering all 47 prefectures from approximately 2.2 million panelists registered in a Japanese Internet survey agency [21] to be representative of the Japanese demographic composition as of October 1, 2019 regarding the distribution of age, sex, and geographic location. A total of 28,000 respondents aged 15 to 79 years participated in the study.

In theory, protective behaviors amid the crisis are influenced by information sources through threat perception [22]. Thus, we used variables on infection preventive behaviors as outcome, information sources as exposure, and threat perception (fear and worry) as potential mediator.

### Participants

Of the 28,000 participants, we excluded 2,518 who provided invalid responses or met other exclusion criteria; these measures to validate the data quality were established previously [20]. Briefly, participants with invalid responses were detected by using a dummy item that stated: “Please choose the second option from the bottom.” The participants who selected an option other than the one indicated were excluded (n = 1,955). Additionally, we excluded those who provided artificial or unnatural responses: 422 participants who reported using all recreational substances and medications, including sleeping pills, legal opioids, illegal opioids, organic solvents, designer drug, cannabis, cocaine, and 141 who reported having all listed chronic diseases, including cancer, ischemic heart disease, stroke, diabetes, asthma, mental disorder, etc. The remaining 25,482 participants (12,809 women and 12,673 men) aged between 15 to 79 years (mean 48.8 [SD: 17.3] years) were included in the main analysis.

### Ethical considerations

This study was performed in accordance with the ethical standards of the 1975 Declaration of Helsinki, as revised in 2013. Online written informed consent was obtained from all participants including minors aged 15 years or older before responding to the online questionnaire. In Japan, minors can be the subject of the consent if the person has completed secondary school and has the ability to participate in the research, and if the ethical committee approved the procedure, according to the ethical guidelines. In the present survey, all participants, aged 15 years or older at the time of the survey, had completed secondary school and the ethical committee approved the inclusion of minors. The study protocol including the procedure of informed consent process for minors was reviewed and approved by the ethics committees at the Osaka International Cancer Institute (no. 20084) and the Teikyo University (no. 20-148-3). The Internet survey agency respected the Act on the Protection of Personal Information in Japan. The data were anonymized and thus researchers do not have access to information that could identify individual participants. The participants were provided a credit point (known as “Epoints”) that could be used for online shopping and cash conversion as an incentive.

### Exposure assessment

Participants reported the types of information sources for obtaining information regarding COVID-19 from the following sources, traditional media: *wide show*, TV news, newspaper, and radio; new media: online news; social networking services: YouTube, Twitter, Facebook, and Instagram; and primary sources of information: local or national government websites. This kind of questionnaire has been used previously [23]. If they utilized a particular source, they further answered its reliability, in other words, trustworthiness, using six response options. Existing studies measured trust in information source similarly [24]. As with existing studies on reliability [25, 26], we dichotomized the degree of reliance into no reliance (not at all reliable/not reliable/not reliable rather than reliable) and reliance (reliable rather than unreliable/reliable/greatly reliable). Subsequently, we classified the participants into three groups based on the use of information source and its reliability: no use of the source, use without reliance, and use with reliance. In addition to TV programs, we treated newspapers, radios, online news, and government websites as exposures. We did not utilize the data on social media because as smaller proportions of the participants used them as reliable COVID-19 information sources: e.g., YouTube 12.9%, Twitter 12.4%, Facebook 5.2%, Instagram 5.2% compared with other sources and we included a variable for online news as new media, that may be partly obtained through social media.

### Outcome assessment

Participants answered their frequency (none, rarely, sometimes, and always) of engaging in each recommended preventive behavior in the previous month: (1) hand washing using soap for 15 seconds or longer, (2) wearing a mask around people, and (3) attempting to maintain a physical distance of two meters. We dichotomized the frequency of engaging in each behavior into always or not. Similar questionnaires have been used amid this pandemic [27]. To detect individuals who engaged in recommended preventive behaviors strictly, we further divided the participants into two groups: one group consists of those who always adhered to all the three preventive behaviors including hand washing, mask wearing, and trying to keep physical distance, and another consists of the rest.

Participants also reported whether they alerted others who did not engage in preventive behaviors such as hand washing, mask wearing, and physical distancing, since April 2020 using “yes” or “no” response options. This questionnaire is similar to existing one [19].

Fear was assessed using the fear of COVID-19 scale [28], validated in Japanese [29]. This scale consists of seven items rated on a 5-point scale, yielding 7 to 35 points. A higher score indicates a greater fear of COVID-19. Due to the absence of an established cutoff point, we used a cutoff point of 28, approximately corresponding to having fear regarding all seven items, to detect excessive fear for an easier interpretation of the results. Additionally, participants reported their worry since April 2020 caused by others’ infection preventive behaviors (e.g., hand washing, mask wearing, and physical distancing) using one item with two response options as yes or no.

### Other variables

Participants reported socio-demographic factors including age, sex, education level, marital status, number of people living together, prefectural level place of residence, annual household income in 2019, and working conditions. We classified the place of residence into three areas based on the timing of the declaration of the state of emergency in May 2020 as follows: declared first in seven prefectures: Tokyo, Kanagawa, Saitama, Chiba, Osaka, Chiba, and Fukuoka, declared secondly in six prefectures: Hokkaido, Ibaraki, Ishikawa, Gifu, Aichi, and Kyoto, and declared last in the rest 34 prefectures. The personality trait of aggressiveness was self-reported using one item with seven response options (totally disagree to totally agree) and dichotomized as aggressive or not. Health literacy was assessed using a validated scale in Japanese [30]. Additionally, participants reported whether they were alerted by others regarding their own infection preventive behaviors with response options as yes or no.

### Statistical analysis

The participant characteristics according to the watching *wide show* were shown as mean (standard deviation) or number (%). We estimated prevalence ratios (PRs) and their 95% confidence intervals (CIs) of behaviors according to information sources using the log-linear prevalence models, fitted by the working Poisson distributional assumption with log link in the generalized linear models accompanied by robust standard errors, also known as the modified Poisson regression technique.

We calculated the PRs of (1) engaging in recommended preventive behaviors strictly (i.e., always engaging in hand washing, mask wearing, and physical distancing) and (2) alerting others, respectively. Model 1 was adjusted for age, sex, education, income, marital status, number of people living together, working status, and residential area. In Model 2, we additionally and mutually adjusted for the use of information sources. For example, for the analysis of *wide show*, we adjusted for TV news (no watching, watching without reliance, and watching with reliance), newspapers, radios, online news, and government websites.

As sensitivity analyses, first, we analyzed the relationship between information sources and each of the preventive behaviors, including hand washing, mask wearing, and physical distancing. We also conducted age- or sex-specific analyses for the associations of information source with (1) behaviors and (2) emotions. Lastly, to assess the potential mediating role of the fear of COVID-19 and worry because of others in the relationship of the information sources with the preventive behaviors and alerting, we performed the following analyses. Note that our analysis was based on cross-sectional data on self-reported psychological/behavioral attitudes, so we did not formally seek the causal mechanisms between the variables (e.g., by mediation analysis). Instead, we did or did not adjust for the threat perception for the association between supposed exposures and outcome as a sensitivity analysis. Thus, the findings were just exploratory and do not provide clinical implications. Two-sided P values less than 0.05 were considered as statistically significant. These analyses were performed using Stata (ver. 14.2, StataCorp).

## Results

The major trustworthy information sources of COVID-19 were TV news (72.4%), followed by online news (54.1%), *wide show* (50.3%), newspapers (44.5%), and national or local government websites (41.5%). Overall, the prevalence of excessive fear was relatively low (6.9%), while worry because of others’ infection preventive behaviors was high (32.9%). The most frequently adopted preventive behavior was wearing mask (86.0% wore a mask always), followed by hand washing using soap (57.7%) and physical distancing (44.7%). 32.8% of participants engaged in all the three recommended behaviors always. One in ten people alerted others about infection preventive behaviors (9.6%).

As shown in Table 1, compared with the individuals who did not watch *wide show*, those who watched it reliably tended to be older, female, married, and used other information sources reliably. They were less likely to have higher education, engage in work, and live alone. Other variables were not materially different.

**Table 1:** Participants characteristics according to the status of watching *wide show* as a COVID-19 information source.

	*Wide show*			Total
	No watching	Watching without reliance	Watching with reliance	
Participants, n	8900	3761	12,821	25,482
Age, years	43.9 (16.9)	49.4 (16.6)	52.0 (17.1)	48.8 (17.3)
Sex				
Men	5012 (56.3%)	1990 (52.9%)	5671 (44.2%)	12,673 (49.7%)
Women	3888 (43.7%)	1771 (47.1%)	7150 (55.8%)	12,809 (50.3%)
Education				
University or higher	4071 (45.7%)	1763 (46.9%)	4938 (38.5%)	10,772 (42.3%)
College, high school, or lower	4829 (54.3%)	1998 (53.1%)	7883 (61.5%)	14,710 (57.7%)
Marital status				
Unmarried	3708 (41.7%)	1128 (30.0%)	2970 (23.2%)	7806 (30.6%)
Married	4423 (49.7%)	2316 (61.6%)	8491 (66.2%)	15230 (59.8%)
Divorced or bereaved	769 (8.6%)	317 (8.4%)	1360 (10.6%)	2446 (9.6%)
Residential area based on the state of emergency in May 2020				
Declared first	4193 (47.1%)	1837 (48.8%)	5556 (43.3%)	11,586 (45.5%)
Declared second	1481 (16.6%)	621 (16.5%)	2237 (17.4%)	4339 (17.0%)
Declared last	3226 (36.2%)	1303 (34.6%)	5028 (39.2%)	9557 (37.5%)
Number of people living together				
0	2218 (24.9%)	702 (18.7%)	2077 (16.2%)	4997 (19.6%)
1	2492 (28.0%)	1347 (35.8%)	4815 (37.6%)	8654 (34.0%)
2	1936 (21.8%)	870 (23.1%)	2908 (22.7%)	5714 (22.4%)
3 or more	2254 (25.3%)	842 (22.4%)	3021 (23.6%)	6117 (24.0%)
Working status				
Currently working	5919 (66.5%)	2326 (61.8%)	7209 (56.2%)	15,454 (60.6%)
Retired	199 (2.2%)	194 (5.2%)	672 (5.2%)	1065 (4.2%)
Student	900 (10.1%)	237 (6.3%)	614 (4.8%)	1751 (6.9%)
Not working except retirement or education	1882 (21.1%)	1004 (26.7%)	4326 (33.7%)	7212 (28.3%)
Annual household income in 2019				
Below 3 million yen	1740 (19.6%)	659 (17.5%)	2299 (17.9%)	4698 (18.4%)
3 million to < 5 million yen	1806 (20.3%)	734 (19.5%)	2991 (23.3%)	5531 (21.7%)
5 million to < 7 million yen	1350 (15.2%)	561 (14.9%)	2053 (16.0%)	3964 (15.6%)
7 million yen or higher	2056 (23.1%)	1051 (27.9%)	2908 (22.7%)	6015 (23.6%)
Unclear or not want to answer	1948 (21.9%)	756 (20.1%)	2570 (20.0%)	5274 (20.7%)
Health literacy				
High (4 to 5 points)	2577 (29.0%)	1217 (32.4%)	4688 (36.6%)	8482 (33.3%)
Not high (<4 points)	6323 (71.0%)	2544 (67.6%)	8133 (63.4%)	17,000 (66.7%)
Aggressive personality				
Yes (slightly agree/agree/totally agree)	1072 (12.0%)	485 (12.9%)	1334 (10.4%)	2,891 (11.3%)
No (neither disagree nor agree/slightly disagree/disagree/totally disagree)	7828 (88.0%)	3276 (87.1%)	11,487 (89.6%)	22,591 (88.7%)
Been alerted by others				
Yes	489 (5.5%)	273 (7.3%)	773 (6.0%)	1535 (6.0%)
No	8411 (94.5%)	3488 (92.7%)	12,048 (94.0%)	23,947 (94.0%)
Other information sources of COVID-19				
TV news				
No watching	4011 (45.1)	84 (2.2)	200 (1.6)	4295 (16.9)
Watching without reliance	1087 (12.21)	1609 (42.8)	45 (0.4)	2741 (10.8)
Watching with reliance	3802 (42.7)	2068 (55.0)	12,576 (98.1)	18,446 (72.4)
Newspaper				
No reading	6037 (67.8)	1714 (45.6)	5462 (42.6)	13,213 (51.9)
Reading without reliance	350 (3.9)	507 (13.5)	61(0.5)	918 (3.6)
Reading with reliance	2513 (28.2)	1540 (40.9)	7298 (56.9)	11,351 (44.5)
Radio				
No listening	7947 (89.3)	2865 (76.2)	9639 (75.2)	20,451 (80.3)
Listening without reliance	110 (1.2)	340 (9.0)	24 (0.2)	474 (1.9)
Listening with reliance	843 (9.5)	556 (14.8)	3158 (24.6)	4557 (17.9)
Online news				
No browsing	4053 (45.5)	679 (18.1)	2727 (21.3)	7459 (29.3)
Browsing without reliance	1453 (16.3)	1874 (49.8)	901 (7.0)	4228 (16.6)
Browsing with reliance	3394 (38.1)	1208 (32.1)	9193 (71.7)	13,795 (54.1)
Government websites				
No browsing	5943 (66.8)	1814 (48.2)	6439 (50.2)	14,196 (55.7)
Browsing without reliance	237 (2.7)	323 (8.6)	163 (1.3)	723 (2.8)
Browsing with reliance	2720 (30.6)	1624 (43.2)	6219 (48.5)	10,563 (41.5)

Data are summarized as mean (SD) or number (%).

Table 2 shows the associations of the COVID-19 information sources with engaging in strict preventive behaviors. Watching *wide show* was not positively associated with strict preventive behaviors after adjustment for other information sources (Model 2, adjusted PR: 0.96), as well as each behavior as shown in S1 Table. Although watching TV news was not associated with engaging in strict preventive behaviors after adjustment of other information sources (Model 2 in Table 2), watching TV news reliably was significantly associated with each of hand washing, wearing masks, and physical distancing (Model 2, S1 Table). Browsing government websites reliably was significantly and positively associated with these recommended behaviors (Model 2 in Table 2 and S1 Table).

**Table 2:** Prevalence ratios of recommended preventive behaviors or alerting others according to the COVID-19 information sources.

		Engaging in recommended preventive behaviors strictly (hand washing, mask wearing, and physical distancing always)					Alerting others not engaging in infection preventive behaviors				
Information sources	*N*	Prevalence, *n* (%)	PR (Model 1)*	P value	PR (Model 2)^†^	P value	Prevalence, *n* (%)	PR (Model 1)*	P value	PR (Model 2)^†^	P value
***Wide show***											
No watching	8900	2684 (30.2%)	1 (reference)		1 (reference)		657 (7.4%)	1 (reference)		1 (reference)	
Watching without reliance	3761	1259 (33.5%)	1.05 (0.99, 1.11)	0.09	1.01 (0.95, 1.07)	0.78	442 (11.8%)	1.78 (1.59, 2.00)	<0.001	1.48 (1.29, 1.69)	<0.001
Watching with reliance	12,821	4412 (34.4%)	1.05 (1.01, 1.09)	0.025	0.96 (0.91, 1.01)	0.081	1355 (10.6%)	1.66 (1.52, 1.82)	<0.001	1.34 (1.20, 1.49)	<0.001
**TV news**											
No watching	4295	1193 (27.8%)	1 (reference)		1 (reference)		306 (7.1%)	1 (reference)		1 (reference)	
Watching without reliance	2741	821 (30.0%)	1.03 (0.95, 1.10)	0.50	0.97 (0.89, 1.06)	0.48	288 (10.5%)	1.59 (1.37, 1.86)	<0.001	1.01 (0.83, 1.22)	0.94
Watching with reliance	18,446	6341 (34.4%)	1.11 (1.05, 1.17)	<0.001	1.03 (0.96, 1.10)	0.40	1860 (10.1%)	1.70 (1.51, 1.91)	<0.001	1.07 (0.92, 1.24)	0.40
**Newspaper**											
No reading	13,213	4019 (30.4%)	1 (reference)		1 (reference)		1215 (9.2%)	1 (reference)		1 (reference)	
Reading without reliance	918	290 (31.6%)	1.09 (0.99, 1.20)	0.093	1.09 (0.98, 1.22)	0.12	118 (12.9%)	1.61 (1.35, 1.92)	<0.001	1.33 (1.09, 1.62)	0.006
Reading with reliance	11,351	4046 (35.6%)	1.13 (1.09, 1.18)	<0.001	1.06 (1.02, 1.11)	0.005	1121 (9.9%)	1.40 (1.29, 1.52)	<0.001	1.11 (1.02, 1.21)	0.02
**Radio**											
No listening	20,451	6474 (31.7%)	1 (reference)		1 (reference)		1839 (9.0%)	1 (reference)		1 (reference)	
Listening without reliance	474	147 (31.0%)	1.05 (0.91, 1.20)	0.51	1.03 (0.90, 1.19)	0.65	68 (14.3%)	1.65 (1.32, 2.05)	<0.001	1.26 (0.98, 1.61)	0.068
Listening with reliance	4557	1734 (38.1%)	1.21 (1.16, 1.27)	<0.001	1.16 (1.11, 1.21)	<0.001	547 (12.0%)	1.56 (1.42, 1.71)	<0.001	1.29 (1.17, 1.41)	<0.001
**Online news**											
No browsing	7459	2255 (30.2%)	1 (reference)		1 (reference)		492 (6.6%)	1 (reference)		1 (reference)	
Browsing without reliance	4228	1350 (31.9%)	1.06 (1.01, 1.12)	0.032	0.99 (0.93, 1.05)	0.69	439 (10.4%)	1.51 (1.34, 1.71)	<0.001	1.09 (0.95, 1.25)	0.22
Browsing with reliance	13,795	4750 (34.4%)	1.12 (1.07, 1.16)	<0.001	1.04 (0.99, 1.09)	0.10	1523 (11.0%)	1.64 (1.49, 1.81)	<0.001	1.26 (1.13, 1.41)	<0.001
**Government websites**											
No browsing	14,196	4153 (29.3%)	1 (reference)		1 (reference)		1029 (7.2%)	1 (reference)		1 (reference)	
Browsing without reliance	723	247 (34.2%)	1.18 (1.07, 1.31)	0.001	1.19 (1.07, 1.32)	0.002	113 (15.6%)	2.14 (1.79, 2.56)	<0.001	1.87 (1.55, 2.27)	<0.001
Browsing with reliance	10,563	3955 (37.4%)	1.24 (1.20, 1.29)	<0.001	1.21 (1.16, 1.25)	<0.001	1312 (12.4%)	1.76 (1.62, 1.90)	<0.001	1.52 (1.40, 1.65)	<0.001

PR, prevalence ratio; TV, television. The data are shown as PRs (95% confidence intervals).

*Adjusted for age, sex, education, marital status, number of people living together, working status, annual income, and residential area.

^†^Adjusted for factors in Model 1 and the other COVID-19 information sources (e.g., for analysis of *wide show*, we adjusted for TV news, newspapers, radio, online news, and government websites).

As shown in Table 2, all information sources, except TV news, were significantly and positively associated with alerting others even after mutual adjustment of information sources (Model 2). For example, watching *wide show* reliably demonstrated 1.34 times higher PR of alerting (95% CI: 1.20 to 1.49) in Model 2. The age- and sex-specific analyses showed similar results to the main ones as shown in S2 and S3 Tables.

Regarding the information sources of COVID-19 and emotions shown in Table 3, overall, except for TV news and online news, the prevalence of excessive fear among individuals who used the information sources was significantly higher compared with those who did not use even after adjustment for other information sources. For example, watching *wide show* reliably was associated with a significantly higher prevalence of excessive fear (the adjusted PR: 1.36 [95% CI: 1.19, 1.54] in Model 2). In contrast, watching TV news and browsing online news reliably were significantly associated with lowered prevalence of excessive fear (the adjusted PRs: 0.62 and 0.87, respectively in Model 2). For analysis of worry because of others, all information sources except for newspaper were significantly linked to a higher prevalence of worry. For example, watching *wide show* reliably was significantly associated with worry (the adjusted PR: 1.20 in Model 2). These results were not largely different by age and sex (S4 and S5 Tables).

**Table 3:** Prevalence ratios of fear or worry because of others’ behaviors according to COVID-19 information sources.

		Excessive fear of COVID-19			Worry because of others’ infection preventive behaviors		
Information sources	N	Prevalence, n (%)	PR (95% CI)*	P value	Prevalence, n (%)	PR (95% CI)*	P value
***Wide show***							
No watching	8900	595 (6.7%)	1 (reference)		1993 (22.4%)	1 (reference)	
Watching without reliance	3761	176 (4.7%)	0.79 (0.66, 0.95)	0.011	1603 (42.6%)	1.38 (1.30, 1.46)	<0.001
Watching with reliance	12,821	979 (7.6%)	1.36 (1.19, 1.54)	<0.001	4777 (37.3%)	1.20 (1.14, 1.26)	<0.001
**TV news**							
No watching	4295	366 (8.5%)	1 (reference)		644 (15.0%)	1 (reference)	
Watching without reliance	2741	149 (5.4%)	0.67 (0.55, 0.83)	<0.001	948 (34.6%)	1.43 (1.29, 1.57)	<0.001
Watching with reliance	18,446	1235 (6.7%)	0.62 (0.53, 0.73)	<0.001	6781 (36.8%)	1.61 (1.48, 1.75)	<0.001
**Newspaper**							
No reading	13,213	883 (6.7%)	1 (reference)		4002 (30.3%)	1 (reference)	
Reading without reliance	918	73 (8.0%)	1.37 (1.06, 1.78)	0.017	297 (32.4%)	0.97 (0.87, 1.08)	0.53
Reading with reliance	11,351	794 (7.0%)	1.03 (0.93, 1.15)	0.53	4074 (35.9%)	1.03 (0.99, 1.07)	0.20
**Radio**							
No listening	20,451	1315 (6.4%)	1 (reference)		6497 (31.8%)	1 (reference)	
Listening without reliance	474	43 (9.1%)	1.68 (1.23, 2.30)	0.001	165 (34.8%)	0.95 (0.83, 1.08)	0.41
Listening with reliance	4557	392 (8.6%)	1.34 (1.19, 1.50)	<0.001	1771 (38.9%)	1.07 (1.02, 1.11)	0.005
**Online news**							
No browsing	7459	581 (7.8%)	1 (reference)		1344 (18.0%)	1 (reference)	
Browsing without reliance	4228	233 (5.5%)	0.78 (0.67, 0.92)	0.002	1737 (41.1%)	1.70 (1.59, 1.81)	<0.001
Browsing with reliance	13,795	936 (6.8%)	0.87 (0.78, 0.97)	0.01	5292 (38.4%)	1.56 (1.47, 1.65)	<0.001
**Government websites**							
No browsing	14,196	941 (6.6%)	1 (reference)		3449 (24.3%)	1 (reference)	
Browsing without reliance	723	67 (9.3%)	1.54 (1.19, 1.98)	0.001	312 (43.2%)	1.57 (1.43, 1.72)	<0.001
Browsing with reliance	10,563	742 (7.0%)	1.10 (1.00, 1.21)	0.049	4612 (43.7%)	1.50 (1.44, 1.55)	<0.001

CI, confidence interval; PR, prevalence ratio.

*Adjusted for age, sex, education, marital status, number of people living together, working status, annual income, residential area, and the other COVID-19 information sources (Model 2).

Regarding the association of emotions with behaviors (S6 Table), both excessive fear and worry were significantly and positively associated with engaging in strict preventive behaviors and alerting. For example, excessive fear was associated with significantly higher prevalence of strict preventive behaviors and alerting (the adjusted PRs: 1.73 and 1.64, respectively in Model 1). However, the adjustment of fear and worry did not materially change the associations of information sources with strict preventive behaviors (Model 3 in S7 Table). Although the adjustment of these emotions attenuated the associations of information sources with alerting to some extent, watching *wide show* and browsing government websites reliably were still significantly and positively linked to alerting (Model 3 in S7 Table).

## Discussion

We found that watching *wide show* as a reliable information source of COVID-19 was significantly and positively associated with alerting others who did not engage in infection preventive behaviors in Japan. However, watching *wide show* did not show such associations with recommended preventive behaviors, such as hand washing and mask wearing. In contrast, watching TV news was not linked to alerting others. Browsing government websites was consistently associated with both recommended preventive behaviors and alerting others. This is the first study to investigate the association of watching *wide show* with recommended preventive behaviors and behaviors toward other people.

We observed that watching *wide show* as reliable information source of COVID-19 was not associated with any of recommended preventive behaviors. Although watching TV news was also not associated with engaging in strict preventive behaviors, it was significantly associated with each recommended behavior as shown in S1 Table. These data suggest that the content or the genre of TV programs may be linked to commonly recommended preventive measures against infections. The present findings are consistent with those from the US, showing that individuals who trust CNN rather than FOX News tended to engage in infection preventive behaviors [31]. The present findings together with the US study [31] suggest that the content or genre of TV news may be differentially associated with adoption of recommended preventive measures.

The present data showed that watching *wide show* was significantly associated with alerting others. A previous study from China [9] also reported that traditional media use, defined as TV, broadcast, and newspaper, was positively associated with intervening behaviors toward others. Contrary, we observed that watching TV news was not linked to alerting others. This finding might support those from the UK [19], showing no association of information sources from TV and radio broadcasters with having had arguments, feeling angry, or falling out with someone. The present study together with existing studies [9, 19] suggest that the method of information transmission can affect behaviors toward others.

We found that, in contrast to TV exposure, using government websites as a reliable COVID-19 information source was consistently associated with a greater adherence to infection preventive behaviors. Our data support the previous observations showing that individuals who accessed governmental information sources for COVID-19 tended to adopt preventive behaviors [4, 5]. These individuals might have perceived more threat, and thus had adhered to such preventive behaviors to protect from infection. Although we found that the associations did not disappear even after adjustment for fear and worry, the findings were limited by the cross-sectional design. Additional longitudinal studies are needed to clarify the role of emotions in the relationship between information sources and preventive behaviors.

It is important to consider the potential mechanisms of behaviors among *wide show* audience in contrast to TV news. Briefly, two distinct features in TV news contents were reported between *wide show* programs and hard news programs [13]. First, the focus of the object is toward others in *wide show* programs, whereas toward the audience themselves in hard news programs [13]. For example, high numbers of retweets about *wide show* programs were observed when the topics were crowded places and no masking of others [13]. In contrast, a high number of retweets about TV news program was observed when the topic was the announcement of commonly recommended measures including hand washing and disinfection that audience can do [13]. Second, among 25 hard or soft TV news programs including *wide show* in Japan, “self-restraint” was more frequently referred in *wide show* programs, whereas few in hard news programs [13]. Exposure to the words relating to “self-restraint” and direction to others in *wide show* programs might have assisted to alert to others who do not adhere to preventive measures, who seem non-“self-restraint”, but might not lead to own preventive measures such as hand washing. Further mass media studies are needed to clarify the plausible mechanisms.

In Japan, problems caused by excessive alerting have been reported, for example, demand to close the shop [32], yelling at other people who did not wear mask for alerting and its counter-violence [33]. Thus, it would be meaningful to discuss public health implications as *wide show* may have a potential to help reduce such avoidable troubles related to alerting, although causal-relationship is unclear. As *wide show* has been pointed out to broadcast COVID-19 sensationally [15], it may be important for *wide show* to broadcast the fact in a non-emotional manner. In light of protective action decision model [22], broadcasting appropriate protective action would be also important for *wide show*. Organizational efforts are warranted for TV stations to review and revise own TV programs in order to mitigate the aggressiveness within the society. As with stress management [34], it may be needed for the public to limit the time spending watching *wide show* if they feel agitated. We also found the positive associations of browsing government websites with recommended behaviors and alerting. As it has been recommended to refer to information on COVID-19 from national or local public health authorities [35], the contents of government websites should be monitored for appropriateness until the end of this pandemic.

Limitations of this study should be clarified. First, because of the nature of cross-sectional observations, the causality cannot be established. It is possible that people who want to criticize others may preferentially have watched *wide show* to support their beliefs [36, 37]. Likewise, the results of the mediation analysis should be interpreted in the context of hypothesis generation rather than hypothesis confirmation due to the cross-sectional nature of the data [38]. Second, the chronological relationship between timing of media use and the emotions and behaviors is unclear. Third, the validity and reproducibility of the assessment of media use including watching TV news and *wide show*, worry, preventive behaviors including hand washing and mask wearing, and alerting are not established as with other studies. Nonetheless, TV news and *wide show* have been distinctively addressed [13]. In addition, the prevalence of media use as a COVID-19 information source, negative emotions, and preventive behaviors are similar to those found in previous Japanese studies [3, 39, 40]. The prevalence of intervening behaviors was also similar to those from UK [19]. Fourth, participants were recruited via internet. Thus, participant characteristics might be different from those recruited via other procedure. Lastly, the participants were Japanese. Caution should be exercised when generalizing the present findings to other countries. Despite these limitations, some strengths in the present study include the use of a large-scale nationwide data from Japan and adjustment for a wide array of potential confounders.

## Conclusion

The present data showed that, while browsing government websites as reliable COVID-19 information source was significantly associated with strictly engaging in the recommended preventive behaviors, watching *wide show* and TV news was not associated with such behaviors. Watching *wide show* was merely associated with alerting others. Exposure to *wide show* may not help promote recommended preventive behaviors. Although the causality is unclear, actions may be needed for TV stations broadcasting *wide show* programs and their sponsors to gain a better understanding of own influences on society in a timely way amid the health emergencies; according to the Broadcasting Act of Japan, broadcasters must not negatively influence public safety or good morals.

## Supporting information

S1 Table Prevalence ratios (95% confidence intervals) of individual infection preventive behavior according to the COVID-19 information sources. (PDF)

S2 Table Age-specific analysis for the associations of information sources of COVID-19 with recommended preventive behaviors or alerting others. (PDF)

S3 Table Sex-specific analysis for the associations of information sources of COVID-19 with recommended preventive behaviors or alerting others. (PDF)

S4 Table Age-specific analysis for the associations of information sources of COVID-19 with fear or worry. (PDF)

S5 Table Sex-specific analysis for the associations of information sources of COVID-19 with fear or worry. (PDF)

S6 Table Prevalence ratios (95% confidence intervals) of recommended preventive behaviors or alerting others according to fear or worry. (PDF)

S7 Table Prevalence ratios (95% confidence intervals) of recommended preventive behaviors or alerting others according to the COVID-19 information sources with adjustment for fear and worry. (PDF)

## References

[1] van Bavel JJ, Baicker K, Boggio PS, Capraro V, Cichocka A, Cikara M, et al. Using social and behavioural science to support COVID-19 pandemic response. Nat Hum Behav. 2020;4(5):460–71. doi: 10.1038/s41562-020-0884-z 32355299

[2] Hutchins HJ, Wolff B, Leeb R, Ko JY, Odom E, Willey J, et al. COVID-19 Mitigation Behaviors by Age Group—United States, April-June 2020. MMWR Morb Mortal Wkly Rep. 2020;69(43):1584–90. doi: 10.15585/mmwr.mm6943e4 33119562 PMC7641002

[3] Machida M, Nakamura I, Saito R, Nakaya T, Hanibuchi T, Takamiya T, et al. Changes in implementation of personal protective measures by ordinary Japanese citizens: A longitudinal study from the early phase to the community transmission phase of the COVID-19 outbreak. Int J Infect Dis. 2020;96:371–5. doi: 10.1016/j.ijid.2020.05.039 32425637 PMC7231496

[4] Sengeh P, Jalloh MB, Webber N, Ngobeh I, Samba T, Thomas H, et al. Community knowledge, perceptions and practices around COVID-19 in Sierra Leone: a nationwide, cross-sectional survey. BMJ Open. 2020;10(9):e040328. doi: 10.1136/bmjopen-2020-040328 32948576 PMC7500298

[5] Pan Y, Fang Y, Xin M, Dong W, Zhou L, Hou Q, et al. Self-Reported Compliance With Personal Preventive Measures Among Chinese Factory Workers at the Beginning of Work Resumption Following the COVID-19 Outbreak: Cross-Sectional Survey Study. J Med Internet Res. 2020;22(9):e22457. doi: 10.2196/22457 32924947 PMC7527164

[6] Ahmed MAM, Siewe Fodjo JN, Gele AA, Farah AA, Osman S, Guled IA, et al. COVID-19 in Somalia: Adherence to Preventive Measures and Evolution of the Disease Burden. Pathogens. 2020;9(9). doi: 10.3390/pathogens9090735 32899931 PMC7560173

[7] Pan Y, Xin M, Zhang C, Dong W, Fang Y, Wu W, et al. Associations of Mental Health and Personal Preventive Measure Compliance With Exposure to COVID-19 Information During Work Resumption Following the COVID-19 Outbreak in China: Cross-Sectional Survey Study. J Med Internet Res. 2020;22(10):e22596. doi: 10.2196/22596 32936776 PMC7546870

[8] Romer D, Jamieson KH. Conspiracy theories as barriers to controlling the spread of COVID-19 in the U.S. Soc Sci Med. 2020;263:113356. doi: 10.1016/j.socscimed.2020.113356 32967786 PMC7502362

[9] Niu Z, Wang T, Hu P, Mei J, Tang Z. Chinese Public’s Engagement in Preventive and Intervening Health Behaviors During the Early Breakout of COVID-19: Cross-Sectional Study. J Med Internet Res. 2020;22(8):e19995. doi: 10.2196/19995 32716897 PMC7474413

[10] Liu PL. COVID-19 Information Seeking on Digital Media and Preventive Behaviors: The Mediation Role of Worry. Cyberpsychol Behav Soc Netw. 2020;23(10):677–82. doi: 10.1089/cyber.2020.0250 32498549

[11] Van Aelst P, Toth F., Castro L., Štětkab V., de Vreese C., Aalberge T., et al. Does a Crisis Change News Habits? A Comparative Study of the Effects of COVID-19 on News Media Use in 17 European Countries. Digit Journalism. 2021;9(9):1208–38.

[12] Video Research Ltd. *Changes in the trends in television watching due to the spread of COVID-19*. 2020 May 22 [Cited 2023 Feb 15]. Available from: https://www.videor.co.jp/press/2020/200522.html.

[13] Takahashi K, Hara Y. How Television Reported COVID–19: Looking at the Correlation between Television and Social Media [Part I] Summarizing the 200 Days of Reports and Posts through Data. NHK Monthly Rep Broadcast Res. 2020;70(12):2–35. doi: 10.24634/bunken.70.12_2

[14] Gollust SE, Fowler EF, Niederdeppe J. Television News Coverage of Public Health Issues and Implications for Public Health Policy and Practice. Annu Rev Public Health. 2019;40:167–85. doi: 10.1146/annurev-publhealth-040218-044017 30633711

[15] Suto Y. *Call on "wide show" again to reflect on their broadcasting about COVID-19*. Ronza. 2020 May 7 [Cited 2021 July 1]. Available from: https://webronza.asahi.com/science/articles/2020050500002.html.

[16] Sakai N. Covid-19 broadcasting, eliminate staging: The Sankei Shimbun. 2020 Apr 19 [Cited 2021 July 1]. Available from: https://www.sankei.com/column/news/200419/clm2004190003-n1.html.

[17] Nanasawa K, Higashiyama K, Takahashi K. How Television Reported COVID–19: Looking at the Correlation between Television and Social Media [Part II] PCR Testing: "Agenda Setting" by Broadcasters and Reactions on Twitter. NHK Monthly Rep Broadcast Res. 2021;71(1):24–60. doi: 10.24634/bunken.71.1_24

[18] Sankei Sports. <Measures against COVID-19> *Misunderstanding about Pachinko-hall has been resolving*. 2020 May 20 [Cited 2021 July 1]. Available from: https://www.sanspo.com/etc/news/20200520/pac20052019000005-n1.html.

[19] Smith LE, Duffy B, Moxham-Hall V, Strang L, Wessely S, Rubin GJ. Anger and confrontation during the COVID-19 pandemic: a national cross-sectional survey in the UK. J R Soc Med. 2020:141076820962068. doi: 10.1177/0141076820962068 33115327 PMC7876655

[20] Matsuyama Y, Aida J, Takeuchi K, Koyama S, Tabuchi T. Dental Pain and Worsened Socioeconomic Conditions Due to the COVID-19 Pandemic. J Dent Res. 2021:220345211005782. doi: 10.1177/00220345211005782 33792422 PMC8138328

[21] Rakuten Insight Inc. [Cited 2021 Oct 1]. Available from: https://in.m.aipsurveys.com.

[22] Lindell MK, Perry RW. The protective action decision model: theoretical modifications and additional evidence. Risk Anal. 2012;32(4):616–32. doi: 10.1111/j.1539-6924.2011.01647.x 21689129

[23] Ali SH, Foreman J, Tozan Y, Capasso A, Jones AM, DiClemente RJ. Trends and Predictors of COVID-19 Information Sources and Their Relationship With Knowledge and Beliefs Related to the Pandemic: Nationwide Cross-Sectional Study. JMIR Public Health Surveill. 2020;6(4):e21071. doi: 10.2196/21071 32936775 PMC7546863

[24] Reynolds RM, Weaver SR, Nyman AL, Eriksen MP. Trust in COVID-19 information sources and perceived risk among smokers: A nationally representative survey. PLoS One. 2022;17(1):e0262097. doi: 10.1371/journal.pone.0262097 35085293 PMC8794215

[25] MacIntyre CR, Nguyen PY, Chughtai AA, Trent M, Gerber B, Steinhofel K, et al. Mask use, risk-mitigation behaviours and pandemic fatigue during the COVID-19 pandemic in five cities in Australia, the UK and USA: A cross-sectional survey. Int J Infect Dis. 2021;106:199–207. doi: 10.1016/j.ijid.2021.03.056 33771668 PMC7985682

[26] Trent M, Seale H, Chughtai AA, Salmon D, MacIntyre CR. Trust in government, intention to vaccinate and COVID-19 vaccine hesitancy: A comparative survey of five large cities in the United States, United Kingdom, and Australia. Vaccine. 2022;40(17):2498–505. doi: 10.1016/j.vaccine.2021.06.048 34218963 PMC8220944

[27] Rattay P, Michalski N, Domanska OM, Kaltwasser A, De Bock F, Wieler LH, et al. Differences in risk perception, knowledge and protective behaviour regarding COVID-19 by education level among women and men in Germany. Results from the COVID-19 Snapshot Monitoring (COSMO) study. PLoS One. 2021;16(5):e0251694. doi: 10.1371/journal.pone.0251694 33979413 PMC8116045

[28] Ahorsu DK, Lin CY, Imani V, Saffari M, Griffiths MD, Pakpour AH. The Fear of COVID-19 Scale: Development and Initial Validation. Int J Ment Health Addict. 2020:1–9. 10.1007/s11469-020-00270-8 PMC7100496 32226353

[29] Wakashima K, Asai K, Kobayashi D, Koiwa K, Kamoshida S, Sakuraba M. The Japanese version of the Fear of COVID-19 scale: Reliability, validity, and relation to coping behavior. PLoS One. 2020;15(11):e0241958. doi: 10.1371/journal.pone.0241958 33152038 PMC7644080

[30] Ishikawa H, Nomura K, Sato M, Yano E. Developing a measure of communicative and critical health literacy: a pilot study of Japanese office workers. Health Promot Int. 2008;23(3):269–74. doi: 10.1093/heapro/dan017 18515303

[31] Zhao E, Wu Q, Crimmins EM, Ailshire JA. Media trust and infection mitigating behaviours during the COVID-19 pandemic in the USA. BMJ Glob Health. 2020;5(10). doi: 10.1136/bmjgh-2020-003323 33037063 PMC7545496

[32] The Yomiuri Shimbun. "*Do not gather children" "Close the shop" a shop had been put up a harassing poster*, *damaged mom-and-pop candy store made a comeback*. 2020 Aug 25 [Cited 2021 Oct 1]. Available from: https://www.yomiuri.co.jp/national/20200825-OYT1T50116/.

[33] RADIO Kansai, Ltd. *A man who had been alerted to "wear mask" and had assaulted said "He was truculent*, *I took up his quarrel"*, *Kobe District Court imposed a sentence to probation "just for once"*. 2022 May 17 [Cited 2022 July 1]. [Available from: https://jocr.jp/raditopi/2022/05/17/428757/.

[34] World Health Organization. Coping with stress during the 2019-nCoV outbreak. 2020. [Cited 2023 Feb 20]. Available from: https://www.who.int/docs/default-source/coronaviruse/coping-with-stress.pdf.

[35] World Health Organization. Coronavirus disease (COVID-19) advice for the public. 2020. [Cited 2021 July 1]. [Available from: https://www.who.int/emergencies/diseases/novel-coronavirus-2019/advice-for-public.

[36] Sears DO, Freedman J.L. Selective exposure to information: a critical review. Public Opinion Quarterly. 1967;31(2):194–213.

[37] Nickerson R. Confirmation Bias: A Ubiquitous Phenomenon in Many Guises. Rev Gen Psychol. 1998;2(2):175–220.

[38] Gelfand LA, Mensinger JL, Tenhave T. Mediation analysis: a retrospective snapshot of practice and more recent directions. J Gen Psychol. 2009;136(2):153–76. doi: 10.3200/GENP.136.2.153-178 19350833 PMC2670477

[39] Shiina A, Niitsu T, Kobori O, Idemoto K, Hashimoto T, Sasaki T, et al. Relationship between perception and anxiety about COVID-19 infection and risk behaviors for spreading infection: A national survey in Japan. Brain Behav Immun Health. 2020;6:100101. doi: 10.1016/j.bbih.2020.100101 32835297 PMC7331545

[40] Muto K, Yamamoto I, Nagasu M, Tanaka M, Wada K. Japanese citizens’ behavioral changes and preparedness against COVID-19: An online survey during the early phase of the pandemic. PLoS One. 2020;15(6):e0234292. 32525881 10.1371/journal.pone.0234292 PMC7289361

