# Supplementary material for: Association between watching wide show as a reliable COVID-19 information source and preventive behaviors: A nationwide survey in Japan
Source: PLoS One. 2023 Apr 11;18(4):e0284371. doi: 10.1371/journal.pone.0284371 (PMC10089324; doi:10.1371/journal.pone.0284371)
Supplement: S1 Table — (PDF) [file pone.0284371.s001.pdf]

S1 Table. Prevalence ratios (95% confidence intervals) of individual infection preventive behavior according to the COVID-19 information sources.

	Hand washing		Mask wearing		Physical distancing	
**Information sources**	PR (95% CI)*	P value	PR (95% CI)*	P value	PR (95% CI)*	P value
Wide show						
No watching	1 (reference)		1 (reference)		1 (reference)	
Watching without reliance	1.06 (1.02, 1.10)	0.002	1.03 (1.01, 1.04)	0.001	0.98 (0.93, 1.03)	0.36
Watching with reliance	1.03 (1.00, 1.06)	0.08	1.01 (1.00, 1.02)	0.20	0.96 (0.93, 1.00)	0.033
**TV news**						
No watching	1 (reference)		1 (reference)		1 (reference)	
Watching without reliance	1.11 (1.05, 1.17)	<0.001	1.23 (1.19, 1.26)	<0.001	1.04 (0.97, 1.11)	0.31
Watching with reliance	1.13 (1.08, 1.17)	<0.001	1.25 (1.22, 1.28)	<0.001	1.06 (1.00, 1.11)	0.039
**Newspaper**						
No reading	1 (reference)		1 (reference)		1 (reference)	
Reading without reliance	1.00 (0.93, 1.07)	0.94	0.97 (0.94, 1.01)	0.14	1.02 (0.94, 1.11)	0.63
Reading with reliance	1.04 (1.02, 1.07)	0.001	1.01 (1.00, 1.02)	0.009	1.04 (1.00, 1.07)	0.031
**Radio**						
No listening	1 (reference)		1 (reference)		1 (reference)	
Listening without reliance	1.02 (0.94, 1.11)	0.62	0.91 (0.87, 0.96)	<0.001	1.02 (0.92, 1.14)	0.69
Listening with reliance	1.15 (1.13, 1.18)	<0.001	1.07 (1.06, 1.08)	0.19	1.14 (1.10, 1.18)	<0.001
**Online news**						
No browsing	1 (reference)		1 (reference)		1 (reference)	
Browsing without reliance	1.09 (1.05, 1.13)	<0.001	1.12 (1.10, 1.14)	<0.001	1.01 (0.96, 1.06)	0.31
Browsing with reliance	1.09 (1.06, 1.12)	<0.001	1.10 (1.09, 1.11)	<0.001	1.04 (1.01, 1.08)	0.039
**Government websites**						
No browsing	1 (reference)		1 (reference)		1 (reference)	
Browsing without reliance	1.05 (0.98, 1.13)	0.15	1.01 (0.98, 1.04)	0.62	1.15 (1.06, 1.25)	0.001
Browsing with reliance	1.15 (1.13, 1.18)	<0.001	1.07 (1.06, 1.08)	<0.001	1.14 (1.11, 1.18)	<0.001

CI, confidence interval; PR, prevalence ratio.

*Adjusted for age, sex, education, marital status, number of people living together, working status, annual income, residential area, and the other COVID-19 information sources (Model 2).

.
