# Supplementary material for: Association between watching wide show as a reliable COVID-19 information source and preventive behaviors: A nationwide survey in Japan
Source: PLoS One. 2023 Apr 11;18(4):e0284371. doi: 10.1371/journal.pone.0284371 (PMC10089324; doi:10.1371/journal.pone.0284371)
Supplement: S2 Table — (PDF) [file pone.0284371.s002.pdf]

S2 Table. Age-specific analysis for the associations of information sources of COVID-19 with recommended preventive behaviors or alerting others.

	Engaging in preventive behaviors strictly (hand washing, mask wearing, and physical distancing always)				Alerting others not engaging in infection preventive behaviors			
	Age <60 years (*n* = 17,342)		Age ≥60 years (*n* = 8140)		Age <60 years (*n* = 17,342)		Age ≥60 years (*n* = 8140)	
**Information sources**	PR (95% CI)*	P value	PR (95% CI)*	P value	PR (95% CI)*	P value	PR (95% CI)*	P value
Wide show								
No watching	1 (reference)		1 (reference)		1 (reference)		1 (reference)	
Watching without reliance	0.98 (0.91, 1.06)	0.63	1.04 (0.94, 1.15)	0.45	1.42 (1.22, 1.66)	<0.001	1.65 (1.19, 2.28)	0.002
Watching with reliance	0.97 (0.91, 1.03)	0.26	0.94 (0.87, 1.02)	0.16	1.36 (1.21, 1.54)	<0.001	1.23 (0.93, 1.63)	0.14
**TV news**								
No watching	1 (reference)		1 (reference)		1 (reference)		1 (reference)	
Watching without reliance	0.97 (0.88, 1.08)	0.61	1.00 (0.83, 1.20)	1.00	1.00 (0.82, 1.22)	1.00	1.09 (0.60, 1.95)	0.78
Watching with reliance	1.00 (0.93, 1.08)	0.90	1.14 (0.99, 1.31)	0.06	1.05 (0.89, 1.23)	0.57	1.18 (0.74, 1.89)	0.48
**Newspaper**								
No reading	1 (reference)		1 (reference)		1 (reference)		1 (reference)	
Reading without reliance	1.11 (0.97, 1.27)	0.12	1.02 (0.84, 1.25)	0.81	1.27 (1.02, 1.58)	0.096	1.53 (0.92, 2.55)	0.10
Reading with reliance	1.11 (1.06, 1.17)	<0.001	0.95 (0.89, 1.02)	0.15	1.16 (1.06, 1.28)	0.002	0.92 (0.75, 1.13)	0.42
**Radio**								
No listening	1 (reference)		1 (reference)		1 (reference)		1 (reference)	
Listening without reliance	1.11 (0.94, 1.30)	0.23	0.86 (0.64, 1.16)	0.32	1.38 (1.07, 1.78)	0.015	0.72 (0.31, 1.70)	0.45
Listening with reliance	1.17 (1.10, 1.24)	<0.001	1.15 (1.08, 1.23)	<0.001	1.29 (1.15, 1.43)	<0.001	1.26 (1.05, 1.52)	0.014
**Online news**								
No browsing	1 (reference)		1 (reference)		1 (reference)		1 (reference)	
Browsing without reliance	0.97 (0.90, 1.05)	0.47	1.00 (0.90, 1.11)	0.96	1.14 (0.98, 1.33)	0.096	0.89 (0.65, 1.23)	0.49

Browsing with reliance	1.01 (0.95, 1.17)	0.78	1.08 (1.01, 1.15)	0.03	1.27 (1.12, 1.44)	<0.001	1.25 (1.01, 1.55)	0.043
**Government websites**								
No browsing	1 (reference)		1 (reference)		1 (reference)		1 (reference)	
Browsing without reliance	1.24 (1.09, 1.27)	0.001	1.06 (0.87, 1.30)	0.57	1.88 (1.53, 2.32)	<0.001	1.89 (1.14, 3.16)	0.014
Browsing with reliance	1.26 (1.20, 1.32)	<0.001	1.12 (1.05, 1.19)	<0.001	1.51 (1.38, 1.66)	<0.001	1.53 (1.26, 1.85)	<0.001

CI, confidence interval; PR, prevalence ratio; TV, television.

*Adjusted for age, sex, education, marital status, number of people living together, working status, annual income, residential area, and the other COVID-19 information sources (Model 2).
