# Supplementary material for: Association between watching wide show as a reliable COVID-19 information source and preventive behaviors: A nationwide survey in Japan
Source: PLoS One. 2023 Apr 11;18(4):e0284371. doi: 10.1371/journal.pone.0284371 (PMC10089324; doi:10.1371/journal.pone.0284371)
Supplement: S4 Table — (PDF) [file pone.0284371.s004.pdf]

S4 Table. Age-specific analysis for the associations of information sources of COVID-19 with fear or worry.

	Excessive fear of COVID-19				Worry because of others' infection preventive behaviors			
	Age <60 years (*n* = 17,342)		Age ≥60 years (*n* = 8140)		Age <60 years (*n* = 17,342)		Age ≥60 years (*n* = 8140)	
**Information sources**	PR (95% CI)*	P value	PR (95% CI)*	P value	PR (95% CI)*	P value	PR (95% CI)*	P value
Wide show								
No watching	1 (reference)		1 (reference)		1 (reference)		1 (reference)	
Watching without reliance	0.75 (0.61, 0.94)	0.012	0.88 (0.63, 1.23)	0.45	1.37 (1.29, 1.47)	<0.001	1.35 (1.20, 1.53)	<0.001
Watching with reliance	1.26 (1.08, 1.48)	0.004	1.55 (1.21, 1.97)	<0.001	1.19 (1.13, 1.26)	<0.001	1.21 (1.09, 1.34)	<0.001
**TV news**								
No watching	1 (reference)		1 (reference)		1 (reference)		1 (reference)	
Watching without reliance	0.77 (0.61, 0.97)	0.025	0.45 (0.28, 0.72)	0.001	1.39 (1.24, 1.54)	<0.001	1.64 (1.27, 2.12)	<0.001
Watching with reliance	0.66 (0.55, 0.79)	<0.001	0.53 (0.39, 0.72)	<0.001	1.56 (1.42, 1.70)	<0.001	1.78 (1.42, 2.23)	<0.001
**Newspaper**								
No reading	1 (reference)		1 (reference)		1 (reference)		1 (reference)	
Reading without reliance	1.42 (1.04, 1.94)	0.025	1.26 (0.78, 2.05)	0.35	0.96 (0.84, 1.08)	0.47	1.00 (0.80, 1.25)	0.98
Reading with reliance	1.18 (1.04, 1.35)	0.013	0.77 (0.65, 0.92)	0.004	1.04 (1.00, 1.09)	0.078	1.02 (0.94, 1.10)	0.66
**Radio**								
No listening	1 (reference)		1 (reference)		1 (reference)		1 (reference)	
Listening without reliance	1.91 (1.34, 2.71)	<0.001	1.12 (0.54, 2.31)	0.75	1.04 (0.91, 1.20)	0.55	0.64 (0.44, 0.93)	0.019
Listening with reliance	1.34 (1.15, 1.57)	<0.001	1.30 (1.10, 1.54)	0.002	1.11 (1.05, 1.17)	<0.001	1.03 (0.96, 1.11)	0.42
**Online news**								
No browsing	1 (reference)		1 (reference)		1 (reference)		1 (reference)	
Browsing without reliance	0.77 (0.61, 0.97)	0.025	0.80 (0.60, 1.06)	0.11	1.68 (1.55, 1.82)	<0.001	1.64 (1.27, 2.12)	<0.001
Browsing with reliance	0.66 (0.55, 0.79)	<0.001	0.86 (0.73, 1.02)	0.081	1.57 (1.46, 1.68)	<0.001	1.50 (1.37, 1.64)	<0.001

**Government websites**								
No browsing	1 (reference)		1 (reference)		1 (reference)		1 (reference)	
Browsing without reliance	1.38 (1.02, 1.87)	0.039	1.88 (1.20, 2.94)	0.006	1.57 (1.41, 1.74)	<0.001	1.59 (1.31, 1.93)	<0.001
Browsing with reliance	0.99 (0.87, 1.11)	0.86	1.31 (1.11, 1.54)	0.001	1.55 (1.48, 1.61)	<0.001	1.38 (1.29, 1.48)	<0.001

CI, confidence interval; PR, prevalence ratio.

*Adjusted for age, sex, education, marital status, number of people living together, working status, annual income, residential area, and the other COVID-19 information sources (Model 2).
