# Supplementary material for: Association between watching wide show as a reliable COVID-19 information source and preventive behaviors: A nationwide survey in Japan
Source: PLoS One. 2023 Apr 11;18(4):e0284371. doi: 10.1371/journal.pone.0284371 (PMC10089324; doi:10.1371/journal.pone.0284371)
Supplement: S6 Table — (PDF) [file pone.0284371.s006.pdf]

S6 Table. Prevalence ratios (95% confidence intervals) of recommended preventive behaviors or alerting others according to fear or worry.

	Engaging in preventive behaviors strictly (hand washing, mask wearing, and physical distancing always)			Alerting others not engaging in infection preventive behaviors		
**Emotions**	Prevalence, *n* (%)	PR (95% CI)*	P value	Prevalence, *n* (%)	PR (95% CI)*	P value
**Fear of COVID-19**						
No excessive fear (*n* = 23,732)	7398 (31.2)	1 (reference)		2,188 (9.2)	1 (reference)	
Excessive fear (*n* = 1750)	957 (54.7)	1.73 (1.65, 1.81)	<0.001	266 (15.2)	1.63 (1.45, 1.84)	<0.001
**Worry because of others' infection preventive behaviors**						
No worry (*n* = 17,109)	5278 (30.8)	1 (reference)		645 (3.8)	1 (reference)	
Worry (*n* = 8373)	3077 (36.7)	1.14 (1.10, 1.19)	<0.001	1809 (21.6)	5.75 (5.27, 6.27)	<0.001

CI, confidence interval; PR, prevalence ratio.

*Adjusted for age, sex, education, marital status, number of people living together, annual income, working status, and residential area (Model 1).
