# Supplementary material for: Association between watching wide show as a reliable COVID-19 information source and preventive behaviors: A nationwide survey in Japan
Source: PLoS One. 2023 Apr 11;18(4):e0284371. doi: 10.1371/journal.pone.0284371 (PMC10089324; doi:10.1371/journal.pone.0284371)
Supplement: S7 Table — (PDF) [file pone.0284371.s007.pdf]

S7 Table. Prevalence ratios (95% confidence intervals) of recommended preventive behaviors or alerting others according to the COVID-19 information sources with adjustment for fear and worry.

	Engaging in preventive behaviors strictly (hand washing, mask wearing, and physical distancing always)		Alerting others not engaging in infection preventive behaviors	
**Information sources**	PR (95% CI)*	P value	PR (95% CI)*	P value
Wide show				
No watching	1 (reference)		1 (reference)	
Watching without reliance	1.01 (0.95, 1.07)	0.85	1.22 (1.07, 1.40)	0.003
Watching with reliance	0.94 (0.89, 0.98)	0.01	1.20 (1.08, 1.33)	0.001
**TV news**				
No watching	1 (reference)		1 (reference)	
Watching without reliance	0.98 (0.90, 1.07)	0.72	0.88 (0.73, 1.05)	0.17
Watching with reliance	1.04 (0.98, 1.11)	0.21	0.87 (0.75, 1.00)	0.06
**Newspaper**				
No reading	1 (reference)		1 (reference)	
Reading without reliance	1.07 (0.96, 1.20)	0.21	1.34 (1.11, 1.63)	0.003
Reading with reliance	1.06 (1.02, 1.11)	0.005	1.09 (1.01, 1.19)	0.035
**Radio**				
No listening	1 (reference)		1 (reference)	
Listening without reliance	1.01 (0.88, 1.17)	0.87	1.26 (0.99, 1.59)	0.06
Listening with reliance	1.14 (1.09, 1.19)	<0.001	1.22 (1.11, 1.33)	<0.001
**Online news**				
No browsing	1 (reference)		1 (reference)	
Browsing without reliance	0.99 (0.93, 1.05)	0.65	0.85 (0.74, 0.96)	0.013
Browsing with reliance	1.03 (0.99, 1.08)	0.14	1.01 (0.91, 1.12)	0.83
**Government websites**				
No browsing	1 (reference)		1 (reference)	
Browsing without reliance	1.14 (1.03, 1.27)	0.014	1.39 (1.15, 1.67)	0.001
Browsing with reliance	1.18 (1.14, 1.23)	<0.001	1.19 (1.10, 1.29)	<0.001

CI, confidence interval; PR, prevalence ratio.

*Adjusted for excessive fear of COVID-19 and worry because of others' infection preventive behaviors in addition to covariates in Model 2 (as Model 3).
